# The yeast guanine nucleotide exchange factor Sec7 is a bottleneck in spatial protein quality control and detoxifies neurological disease proteins

**DOI:** 10.1038/s41598-023-41188-0

**Published:** 2023-08-28

**Authors:** Roja Babazadeh, Kara L. Schneider, Arthur Fischbach, Xinxin Hao, Beidong Liu, Thomas Nystrom

**Affiliations:** 1 Institute for Biomedicine, Sahlgrenska Academy, Centre for Ageing and Health – AgeCap, University of Gothenburg, 405 30 Gothenburg, Sweden; 2 Department of Chemistry and Molecular Biology, University of Gothenburg, Medicinaregatan 9 C, 413 90 Gothenburg, Sweden

**Keywords:** Cell biology, Microbiology, Molecular biology

## Abstract

ER-to-Golgi trafficking partakes in the sorting of misfolded cytoplasmic proteins to reduce their cytological toxicity. We show here that yeast Sec7, a protein involved in proliferation of the Golgi, is part of this pathway and participates in an Hsp70-dependent formation of insoluble protein deposits (IPOD). Sec7 associates with the disaggregase Hsp104 during a mild heat shock and increases the rate of Hsp104 diffusion in an Hsp70-dependent manner when overproduced. Sec7 overproduction increased formation of IPODs from smaller aggregates and mitigated the toxicity of Huntingtin exon-1 upon heat stress while Sec7 depletion increased sensitivity to aẞ42 of the Alzheimer’s disease and α-synuclein of the Parkinson’s disease, suggesting a role of Sec7 in mitigating proteotoxicity.

## Introduction

The protein quality control (PQC) machinery in the cell is crucial to keep protein synthesis, folding, transport, degradation and sequestration in a functional balance (proteostasis) and prevents toxicity caused by aberrant proteins. It consists of many concerted pathways, which are largely facilitated by the action of molecular chaperones. There is a multitude of stressors that can cause an imbalance in proteostasis, leading to loss or gain of function of proteins and disrupting cellular processes^1–4^. Such stressors are for example heat shock and oxidative stress. In addition, a breakdown in proteostasis plays a central role in many age-associated neurodegenerative diseases, including Huntington’s, Alzheimer’s and Parkinson’s disease^5–8^; diseases characterized by the accumulation of toxic protein oligomers and aggregates.

Neurodegenerative diseases, breakdown of PQC, and protein aggregation have been linked to defects in intracellular vesicle trafficking occurring during aging in various organisms^9–17^: For example, the Huntingtin protein interacts with proteins involved in vesicle trafficking^18,19^ and the disease protein causes defects in endocytosis^12^. Similarly, aß processing from the amyloid precursor protein (APP) is facilitated by endolysosomal trafficking and is thus directly linked to different endomembrane systems that malfunction in Alzheimer’s disease^10,11,17,20–22^. Moreover, α-synuclein of the Parkinson’s disease is known to mediate membrane trafficking and failures in such trafficking play an important role in α-synuclein aggregation and in cell-to-cell disease propagation^16,23–27^. In *S. cerevisiae*, there is also evidence that functional membrane trafficking is important to handle α-synuclein aggregates^26,28^.

In addition to the function of endomembrane trafficking in neurological diseases, a role for endocytotic trafficking in yeast aging, proteostasis, and lifespan control has been described previously^29^. Yeast vesicle trafficking is known to consist of several highly conserved interconnected pathways^30–32^, which require constant communication and exchange of material. The systems are thus in close proximity and in contact with one another via cellular organelles and connecting proteins. These trafficking routes consist of the exocytosis/secretory (SEC) pathway, which directs proteins towards the plasma membrane or out of the cell, and the VPS and ALP pathways guiding cargo towards the vacuole. Endocytosis (END pathway) allows for uptake of plasma membrane proteins and extracellular medium components, which after internalization into endosomes are sorted and sent to the vacuole for degradation or to the Golgi for recycling (RCY pathway). The pathways participating in endomembrane trafficking rely on a multitude of factors including Rab GTPases and SNARE proteins such as the yeast syntaxin-5 Sed5, which is involved in COPII anterograde trafficking from the ER to Golgi and was recently shown to function as a bottleneck in spatial PQC (sPQC) in yeast^33^.

The sPQC sorts misfolded proteins into distinct organelle-associated sites within the cell. Upon heat shock, misfolded proteins in the yeast cytoplasm first accumulate at multiple sites called CytoQs, also known as Q-bodies or stress foci, which subsequently coalesce into larger deposition sites commonly referred to as inclusions^34–37^. These inclusions are localized to at least three distinct sites known as the juxtanuclear and/or intranuclear quality control site (JUNQ/INQ); the peripheral, vacuole-associated insoluble protein deposit (IPOD); and a site adjacent to mitochondria^33,35,38–41^.

In this paper, we report that the yeast guanine nucleotide exchange factor, Sec7, a key component in Golgi trafficking^42,43^ is an additional bottleneck in Hsp70-dependent sPQC, including the formation of organelle-associated inclusions, and that overproduction of Sec7 boosts spatial proteostasis during mild heat shock and aging and mitigates the toxicity of Huntingtin exon-1.

## Results and discussion

### Sec7 is a limiting factor in the formation of IPOD inclusions

**Figure 1:**
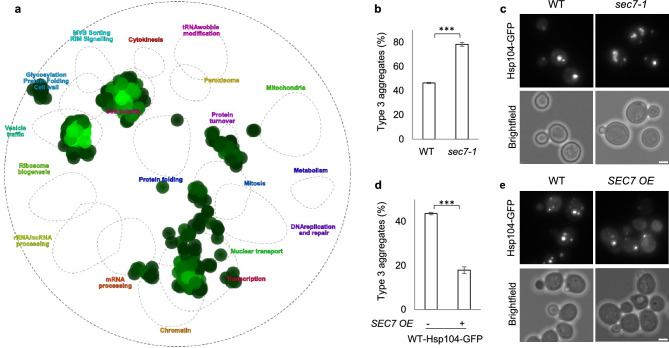
Sec7, among other vesicle trafficking components, was identified to maintain proper spatial PQC upon heat shock. (**a**) SAFE analysis, image modified from TheCellMap.org, hits for type 3 cells upon continuous heat shock for 110 min using the essential gene yeast mutant library. (**b**) Fraction of type 3 in WT, and *sec7-1* mutant cells upon continuous heat shock (38 °C, 90 min). (**c**) Aggregate phenotype of WT and *sec7-1* cells (38 °C, 90 min), representative cells from (**b**). (**d**) Fraction of type 3 upon continuous heat shock (38 °C, 90 min) in WT cells overproducing *SEC7* compared to the vector control (using 316-SEC7-mRFP plasmid (*ADH1* promoter)). (**e**) Aggregate phenotype of WT cells without and with overproduction of Sec7 (38 °C, 90 min, representative cells from (**d**). Images are maximum Z-projections of relevant slices with brightness/contrast adjustment consistently across the strains within the figure. Scale bar 2 μm.

In a recent study, we uncovered the role of vesicle trafficking and specifically the yeast syntaxin-5 in ensuring proper sPQC upon mild heat shock by screening the library of yeast mutants deleted for non-essential genes for defects in inclusion formation^33^. Here, we applied a similar screen including the essential genes of yeast, i.e. performing high content-microscopy after a mild heat shock and determining which temperature sensitive (ts) mutant strains show three or more Hsp104-GFP aggregates per cell (type 3 mutant) rather than the typical one to two inclusions. The main functional categories enriched among the essential genes involved in inclusion formation (SAFE analysis^44^; Fig. 1a) are cell polarity, vesicle trafficking, chromatin/transcription, and protein turnover (Fig. 1a). The role of cell polarity in sPQC has been studied in detail previously^45–47^. Vesicle trafficking was also enriched as a functional category among hits when screening the non-essential mutant library^33^ and confirms this category as involved in sPQC of endogenous protein aggregates upon heat shock. Among the identified genes were known factors required for proper sPQC such as the essential syntaxin-5 and components of the COG complex (supplementary table 1^33^,). In addition to analyzing the data from heat shock after 110 min as an endpoint, we set out to find ts alleles, which cause prolonged failure in aggregate coalescence by monitoring the number of aggregates per cell also at later time points, 220 and 330 min. By comparing the late (330 min) to the early time point (110 min) of heat shock, we pinpointed mutations that caused a drastic failure in sPQC (supplementary table 2).

The essential *SEC7*, with a role in Golgi maturation/proliferation and intra-Golgi trafficking, was selected for further analysis among the hits due to its type 3 phenotype and since it was previously found as a physical interactor of Hsp104 at permissive temperature and even more so during a mild heat shock^29^. Conditional *sec7-1* mutant cells displayed severe defects in the formation of IPOD inclusions and instead harbored multiple (three or more), smaller aggregates (Fig. 1b,c) and only reduced the number of Hsp104-GFP foci-free cells during continued heat shock by 17%. It was thus among the stronger hits of mutants with compromised ability to alleviate Hsp104-associated aggregation during prolonged heat stress. We tested if Sec7 is limiting for IPOD formation by overproducing it on a plasmid (Fig. 1d,e) and by exchanging its native promoter to the strong, constitutive, *GPD* promoter (supplementary Fig. 1a). We found that such overproduction drastically increased IPOD formation from small aggregates, i.e. reduced the number of type 3 cells, indicating that Sec7 is limiting in wild type cells for sPQC. A link between Sec7 and secretion in sPQC was further confirmed by treating cells with brefeldin A, an inhibitor of Sec7 and other secretion factors^33,48,49^ (supplementary Fig. 1b). To determine if Sec7 and Sed5 act through the same pathway in sPQC, we performed aggregate clearance assays with the Sec7 overproduction strain (supplementary Fig. 1c). In contrast to the stark increase in clearance observed in *SED5* overexpression cells^33^, no such effect was found when overexpressing *SEC7*, indicating that overproduction of each of the two factors may not enhance the same sPQC pathways, or that quantitatively different thresholds for achieving an effect in aggregate clearance exist for the two proteins. In addition, we overproduced, via plasmids, Sec7 in *sed5-1* and Sed5 in *sec7-1* conditional mutant cells and found that the defects observed in inclusion formation in each mutant could not be suppressed by such overproduction (supplementary Fig. 1d). This suggests that Sed5 and Sec7 are not functioning in parallel redundant pathways in boosting sPQC as they depend on the presence of each other for their function. Another explanation would be that Sed5 and Sec7 work together to increase inclusion formation.

Next, we wondered if the beneficial effects of Sec7 overproduction on inclusion formation could be explained by its essential role in autophagy^50^, as autophagy contributes to degradation of aberrant proteins. To address this, we tested if the increase in inclusion formation displayed by Sec7 overproduction cells depends on presence of the essential autophagy factor Atg1. *SEC7* overexpression was able to boost inclusion formation also in *atg1∆* background (supplementary Fig. 1e). Additionally, we visualized vacuole morphology in WT, *sec7-1* and *SEC7* overexpressing cells containing Vph1-GFP before and after heat shock. In line with previous data^51^, vacuole area in WT cells increased during heat shock with a simultaneous decrease in vacuole number. While the *sec7-1* conditional mutant displayed an increase in number of small vacuoles upon heat shock, indicating defects in vacuolar dynamics, the Sec7 overproduction strain was unaffected (supplementary Fig. 1f). Collectively, the data indicate that Sec7 action in sPQC is independent of its function in autophagy.

### Sec7 increases the dynamic movement of the disaggregase Hsp104

**Figure 2:**
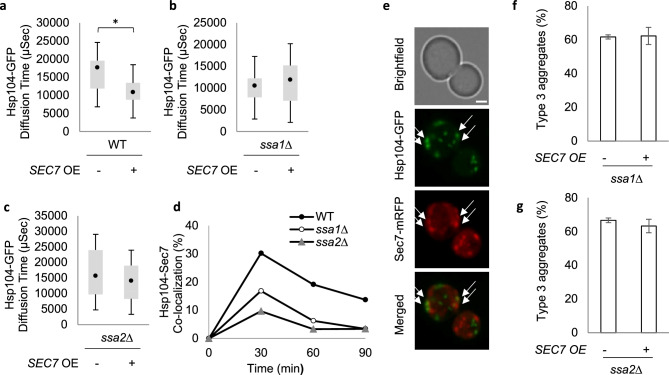
Sec7 overproduction increases the dynamic movement of Hsp104 in a Hsp70-dependent manner. (**a**) Diffusion time of soluble Hsp104-GFP in WT cells without and with overproduction of Sec7 (38 °C). (**b**) Diffusion time of soluble Hsp104-GFP in *ssa1*Δ cells without and with overproduction of Sec7 (38 °C). (**c**) Diffusion time of soluble Hsp104-GFP in *ssa2∆* cells without and with overproduction of Sec7 (38 °C). For (**a**–**c**) The bottom and top of the box represent the first and third quartiles. The circle shows the mean and whiskers indicate the variability of diffusion times outside the upper and lower quartiles for 15–25 cells. (**d**) Fraction of cells displaying co-localization of Hsp104-GFP with Sec7-RFP upon heat shock in WT cells (38 °C). Cells with a visible signal in both channels (green and red) were scored for co-localization. (**e**) Co-localization of Hsp104-GFP with Sec7-RFP after 30 min of heat shock in WT cells. (**f**) Fraction of type 3 upon continuous heat shock (38 °C, 90 min) in *ssa1∆* cells overexpressing *SEC7* compared to the vector control. (**g**) Fraction of type 3 upon continuous heat shock (38 °C, 90 min) in *ssa2*Δ cells overexpressing *SEC7* compared to the vector control.

It has been demonstrated previously that elevated IPOD formation is associated with increased movement of the disaggregase Hsp104 (decreased time of diffusion ^33^;), which specifically binds to protein aggregates and is required for their proper disaggregation^52–54^. Therefore, we investigated if Sec7 overproduction affected Hsp104 diffusion using fluorescence correlation spectroscopy (FCS). We found that the diffusion time was reduced when Sec7 was overproduced (Fig. 2a). Moreover, this effect of Sec7 on Hsp104 movement required the presence of either one of the major cytosolic Hsp70 chaperones, Ssa1 and Ssa2 (Fig. 2b,c), which is interesting as Hsp70s are required for the functions of Hsp104 in protein quality control^55,56^. Sec7 forms foci on Golgi-associated coated vesicles^57^ and we found that such Sec7 foci and Hsp104-associated aggregates transiently co-localized upon mild heat shock^33^ and that this co-localization was dependent on both Ssa1 and Ssa2 (Fig. 2d,e, supplementary Fig. 2). In addition, Ssa1 or Ssa2 was required for Sec7 overproduction to increase IPOD formation from smaller aggregates (Fig. 2f,g).

### Sec7 overproduction reduces aggregation and the presence of multiple aggregates during aging

**Figure 3:**
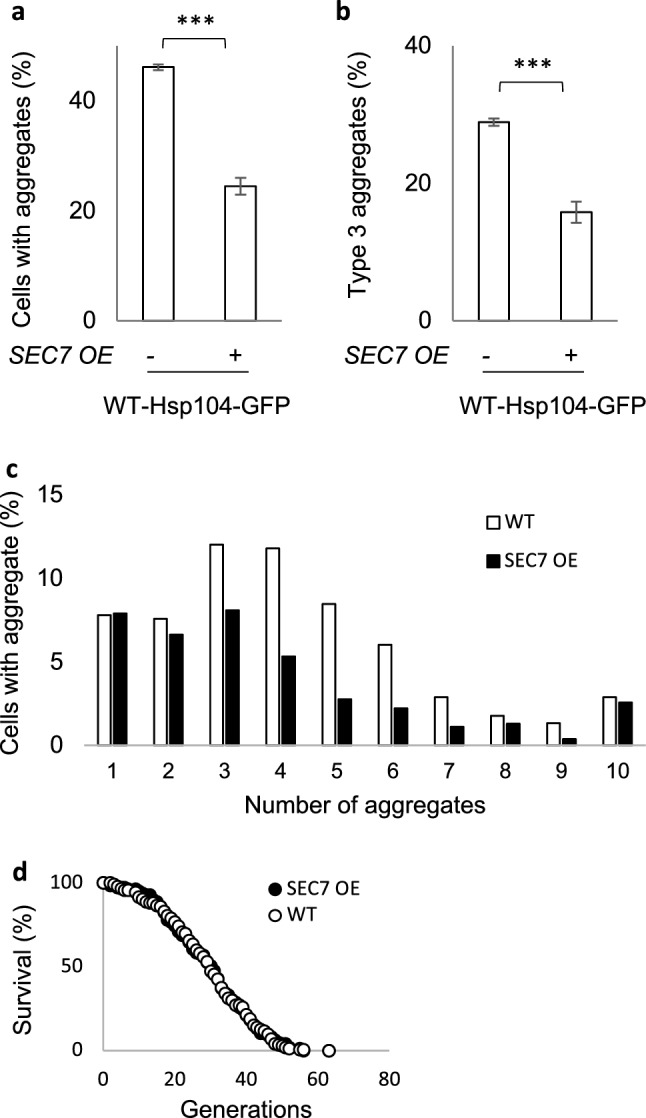
Sec7 overproduction boosts spatial PQC during replicative aging. (**a**) Fraction of aged cells displaying Hsp104-GFP aggregates in WT cells without and with overproduction of Sec7. (**b**) Fraction of type 3 cells in WT without and with overproduction of Sec7. (**c**) Number of aggregates per aged cell of the WT (counted in 449 cells), and *SEC7 OE* strains (counted in 544 cells). (**d**) Replicative lifespan of WT, and *SEC7 OE*.

Since Sec7 appeared limiting for inclusion formation during a mild heat shock, we next investigated if the same was true upon replicative aging. Protein aggregates have been shown to accumulate during replicative aging of yeast mother cells^58,59^ and have a tendency to form multiple aggregates rather than discrete inclusions in old mother cells^60,61^. We isolated old mother cells (average 13 bud scars) and compared their aggregate content with and without Sec7 overproduction. We found, first, that Sec7 overproduction reduced the number of mother cells displaying any type of aggregate (Fig. 3a), and secondly, that old cells that harbored aggregates displayed fewer aggregates when Sec7 was overproduced (Fig. 3b,c). However, we did not find any effect of Sec7 overproduction on the lifespan of cells (Fig. 3d).

### Sec7 affects sensitivity towards human disease proteins

**Figure 4:**
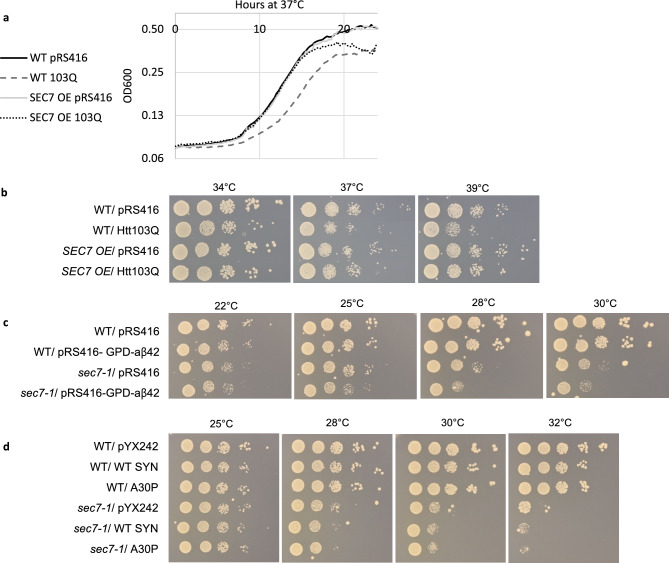
Sec7 affects toxicity of neurodegenerative disease proteins. (**a**) Growth profile in liquid media of WT and *SEC7 OE* cells overproducing Htt103Q via the *GPD* promoter (103Q) compared to the vector control (pRS416) measured using an automated plate reader. (**b**) Fitness of WT and *SEC7 OE* cells overproducing Htt103Q via the *GPD* promoter (Htt103Q) compared to the vector control (pRS416). (**c**) Fitness of WT and *sec7-1* mutant cells overproducing aβ42 (pRS416-GPD- aβ42) compared to the vector control (pRS416). (**d**) Fitness of WT and *sec7-1* mutant cells in the absence (pYX242) or presence of α-synuclein (WT SYN, A30P).

The Huntingtin exon-1, Htt103Q poly-glutamine peptide, of the Huntington disease has been shown to be toxic also in yeast cells^18,62,63^. Given the effect of Sec7 on sPQC, we tested if Sec7 overproduction affected Htt103Q toxicity and found this to be the case both in liquid and plate growth assays (Fig. 4a,b). The liquid growth profile shows that Sec7 overproduction rescues the growth rate during the exponential phase of growth to vector control levels at an elevated temperature, even if the *SEC7* overexpression cells do not reach the same yield as the vector control when monitoring growth for 24 h (Fig. 4a). Additionally, Sec7 overproducing cells display an increased cellular fitness compared to wild type cells containing Htt103Q in growth assays on plates, especially at higher temperatures (Fig. 4b), indicating that overproduction of Sec7 markedly reduces the toxicity of this disease protein. However, *sec7-1* cells are not more sensitive to Htt103Q expression than WT cells (supplementary Fig. 3a). We did not observe any obvious changes in aggregation of Htt103Q in the *SEC7* overexpression cells or in the *sec7-1* conditional mutant compared to WT (supplementary Fig. 3b).

We tested several constructs of two other human disease proteins, aβ42 and α-synuclein of the Alzheimer’s and Parkinson’s disease, respectively. Firstly, we used variants of the proteins that are not, or only very modestly, toxic in yeast cells^17,33^ and found that cells with reduced Sec7 activity, in contrast to wild type cells, displayed sensitivity towards both these proteins (Fig. 4c,d). Secondly, additional constructs of these disease proteins with known toxicity in wild type cells were tested in *SEC7* overexpression cells. α-synuclein and its point mutation A53T version are both toxic when constitutively expressed at high levels via 2μ plasmids^64^ and we found that Sec7 overproduction could not ameliorate toxicity of these constructs and did not affect aggregation of the model proteins (supplementary Fig. 3c,d). Similarly, we used *GAL*-inducible constructs of wild type aβ42, which is moderately toxic, and its highly toxic point mutant version, G37C^65^. Increased levels of Sec7 were unable to mitigate toxicity when disease protein expression was induced by growing cells on galactose (supplementary Fig. 3e). We tested if overexpression of *SEC7* could mitigate any potentially arising toxicity of constitutively overexpressed aβ42 during cellular aging in stationary phase cultures. However, no difference was observed between WT and Sec7 overproducing cells containing aβ42, in contrast to those expressing Htt103Q (supplementary Fig. 3f). We speculate that increased Sec7 levels may be unable to aid α-synuclein and aβ42 detoxification as both these disease proteins interact with, and impede, the secretory pathway^64,65^, which is the pathway Sec7 is expected to boost when overproduced. In contrast to these disease proteins, mutant Huntingtin may not inhibit secretion in such a manner, allowing Sec7 overproduction to act on the pathway and provide resistance against this protein.

In conclusion, recent data has highlighted the role of ER to Golgi trafficking in directing misfolded proteins to the surface of vacuoles and mitochondria and that vesicles of the trafficking pathway may serve as platforms on which misfolded and aggregated proteins can hitchhike towards specific deposition sites^29,33,66^ including IPODs. Such deposition of aggregates at the surface of mitochondria speeds up their clearance from the cell and this deposition also relies on proteins known to make contacts sites between vacuoles and mitochondria^33^. In addition, it is known that deficiencies in endocytosis, and vesicle trafficking in general, renders cells more sensitive to damaged proteins, including neurological disease proteins^12,19^. Herein, we demonstrate that Sec7, a guanine-nucleotide exchange factor required for maturation and proliferation of the Golgi as well as intra-Golgi transport, is a key factor in directing misfolded proteins to the IPOD deposition site. Moreover, Sec7 appears to be a limiting factor in such sPQC as ectopically elevating the levels of the protein is not only boosting IPOD formation but is also mitigating the toxicity of Huntingtin exon-1 during heat stress. It remains to be investigated whether Sec7 Golgi-related functions are mechanistically responsible for the observed effects on sPQC or whether Sec7 may act on sPQC via other pathways. Thus, Sec7, and associated factors involved in Golgi maintenance, might be interesting targets for therapeutic intervention of age-related proteopathies.

## Materials and methods

### Yeast *Saccharomyces cerevisiae*

As described previously^33^, strains with *HSP104-GFP-LEU2* in the S228C SGA (synthetic genetic array) background were used in the inclusion body formation screen as well as in manual aggregate assays and stem from a cross performed according to SGA technology^67^ between the Y7092 SGA query strain with *HSP104-GFP-LEU2* and the essential gene yeast library version 5 unless indicated that the BY4741 background was used. The *SEC7* overexpression in BY4741 was generated using pYM-N15^68^. *ATG1* was deleted using pFA6-hphNT1^68^. Vph1-GFP-HIS3 C-terminal region was amplified from genomic DNA of the GFP collection strain^69^ via PCR and integrated into the WT, *sec7-1* and *SEC7 OE* genome. Detailed information about strains and plasmids are listed in supplementary table S3.

Yeast cells were grown in synthetic drop out media supplemented with 2% glucose and corresponding antibiotics or in YPD. Cells were cultured at 30 °C except for temperature sensitive strains, which were kept at 22 °C.

### High content-microscopy screen

A genome-wide SGA screen was performed as described previously^67^, using a BM3-BC colony handling robot (S&P Robotics Inc.).

Cells from the temperature sensitive mutant collection version 5 (Boone lab, Toronto, Canada) were crossed with the Y7092 query strain containing *HSP104-GFP-LEU2*. The new array was grown in synthetic complete media to an OD_600_ ~ 0.5 at 22 °C and subsequently shifted to 38 °C in a water bath. Cells were collected and imaged live at time points 110 and 330 min of heat shock using the ImageXpress Micro XLS system (Molecular Devices) with a 100 × objective (CFI L Plan EPI cc 0 mm to 0.7 mm) and GFP filter (excitation 472/30 nm, emission 520/35 nm, dichroic mirror 495 nm). The images were analyzed for number of cells and number of aggregates using an algorithm built with MetaXpress software (version 6.54.532, Molecular Devices), which segments the images and keeps masks of shapes to exclude areas outside of cells when determining aggregate number. Mutants were considered hits when cells contained an average of 3 or more Hsp104-GFP aggregates per cell (type 3 phenotype) after 110 min heat insult. The hits were analyzed for functional enrichments (SAFE^44^) using the in-browser tool TheCellMap.org ^70^ by uploading the gene list of hits and setting the significance cutoff to 5e-2. Mutants with prolonged failure in sPQC were identified by determining the percentage of aggregates per cell detectable after 330 min of heat shock in comparison to the initial 110 min time point.

### Protein aggregation analysis

Cells were grown in respective media supplemented with 2% glucose at permissive temperature (30 °C/22 °C) until mid-exponential phase (OD_600_ ~ 0.5). Protein aggregation was induced by shifting the cells to 38 °C for 90 min. Cells were fixed in 3.7% final concentration formaldehyde and washed with PBS. Z-stack images were acquired using a conventional fluorescence microscope, Zeiss Axio Observer .Z1 inverted microscope equipped with Axiocam 506 camera, and a Plan-Apochromat 100x/1.40 NA Oil DIC M27 objective. Images were quantified by manually counting fraction of type 3 cells (containing three aggregates or more) in ImageJ software. Vph1-GFP containing cells and those expressing disease protein plasmids were imaged directly without formaldehyde fixation.

All figure data are based on the average of at least three individual experiments, error bars representing SD. Data were tested for significance using an unpaired two-tailed t-test, with p-values < 0.05 considered significant, depicted as * < 0.05, ** < 0.005, *** < 0.0005.

### Clearance assay

Aggregate clearance assay was performed as described previously^33^.

### Brefeldin A treatment

The treatment was performed as described previously^33^.

### Fluorescence correlation spectroscopy

Fluorescence correlation spectroscopy (FCS) was performed as described previously^33^. In brief, measurements were performed on a ConfoCor2 FCS unit attached to an inverted LSM 880 confocal microscope (Zeiss) equipped with a C-Apochromat 40x/1.20 W Corr objective. To find the measurement position in the cytoplasm of each cell, GFP (excited at 488 nm, emission detected at 500–530 nm) and the transmission channel were simultaneously imaged using an open pinhole. Measurements were performed for 10 × 8 s with excitation at 488 nm and detection at 500–550 nm, pinhole 1 AU. Cells were grown until mid-exponential phase (OD_600_ ~ 0.5), heat shocked for 20 min at 38 °C and attached to ConA treated glass bottom culture dishes while the incubation chamber was at 38 °C. 15–25 cells were analyzed in each condition. 488 nm laser power was set to an AOTF transmission of 0.005%. Fitting of the FCS autocorrelation curves was done using a single component free diffusion fit in the ConfoCor2 software.

### Co-localization analysis

Cells with indicated reporters were grown in respective media supplemented with 2% glucose at permissive temperature (30 °C/22 °C) until mid-exponential phase (OD_600_ ~ 0.5). Protein aggregation was then induced at 38 °C. Co-localization was investigated during or post heat shock at the depicted time points.

### Replicative lifespan analysis

The replicative lifespan analysis was performed as described previously^71^. In brief, early logarithmic phase cells were plated and virgin cells were arranged according to a grid on YPD plates using the micromanipulator MSM400 (Singer Instruments). These cells were the designated new mother cells whose daughter cells were continuously removed and scored. The plates were incubated at 30 °C and cells were dissected at room temperature until they ceased dividing. Overnight, plates were kept at 4 °C. Each analysis was performed independently two times.

### Growth assay on plates

Strains with indicated plasmids were grown in respective media supplemented with 2% glucose at permissive temperature (30 °C/22 °C) until mid-exponential phase (OD_600_ ~ 0.5). The culture ODs were adjusted to 0.5 and serial dilutions at a factor of 10 were performed in multiwell plates. The cell suspensions were spotted onto the respective drop out media plates and incubated at the indicated temperatures for 2 days except when noted otherwise.

### Stationary phase growth assay

Strains with indicated plasmids were grown in -Ura drop out media supplemented with 2% glucose at permissive temperature (30 °C/22 °C) overnight and re-diluted in 15 ml fresh media to OD_600_ = 0.1. The cultures were kept at 30 °C for growth into stationary phase. After 3 days and 10 days of growth, 150 μl were sampled from the cultures, serially diluted at a factor of 10 and spotted onto the respective drop out media plates. Plates were incubated at 30 °C for 2 days and imaged.

### Liquid growth assay

Cells were grown at permissive temperature, 22 °C until reaching logarithmic phase. They were re-diluted to an OD_600_ = 0.01 and arranged as 2–3 technical replicates (error bars are SD) on 24 well-plates. The plate was incubated at 37 °C in the BioTek Synergy 2 plate reader for 24 h with fast shaking while taking measurements of the OD_600_ every 15 min.

### Old cell isolation

Old cells were isolated using the magnetabind biotin-streptavidin method^29,72,73^. Biotin labeled cells were isolated after growing overnight until OD_600_ < 1. The average replicative age of the cells was determined by manually counting bud scars after fixing cells in 3.7% formaldehyde (final concentration) and staining with Wheat Germ Agglutinin Alexa Fluor 555 conjugate (10 mg/ml final concentration, Life Technologies).

### Supplementary Information

Supplementary Information.

## Data Availability

All data generated or analyzed during this study are contained in this article and its supplementary data files. Requests for strains and materials should be directed to the corresponding author.
