# Supplementary material for: The yeast guanine nucleotide exchange factor Sec7 is a bottleneck in spatial protein quality control and detoxifies neurological disease proteins
Source: Sci Rep. 2023 Aug 28;13:14068. doi: 10.1038/s41598-023-41188-0 (PMC10462735; doi:10.1038/s41598-023-41188-0)
Supplement: Supplementary file 1 — Supplementary Information. [file 41598_2023_41188_MOESM1_ESM.pdf]

The yeast guanine nucleotide exchange factor Sec7 is a bottleneck in spatial protein quality control and detoxifies neurological disease proteins.

Roja Babazadeh[1,2], Kara L. Schneider[1,2], Arthur Fischbach[1], Xinxin Hao[1], Beidong Liu[3] and Thomas Nystrom[1,*].

[1] Institute for Biomedicine, Sahlgrenska Academy, Centre for Ageing and Health – AgeCap, University of Gothenburg, Gothenburg, 405 30, Sweden

[2] These authors contributed equally

[3] Department of Chemistry & Molecular Biology, University of Gothenburg, Medicinaregatan 9 C, 413 90 Gothenburg, Sweden

* Lead contact, correspondence to thomas.nystrom@cmb.gu.se

Supplementary material:
Supplementary figures 1-3
Supplementary tables S1-3

Supplementary figure 1

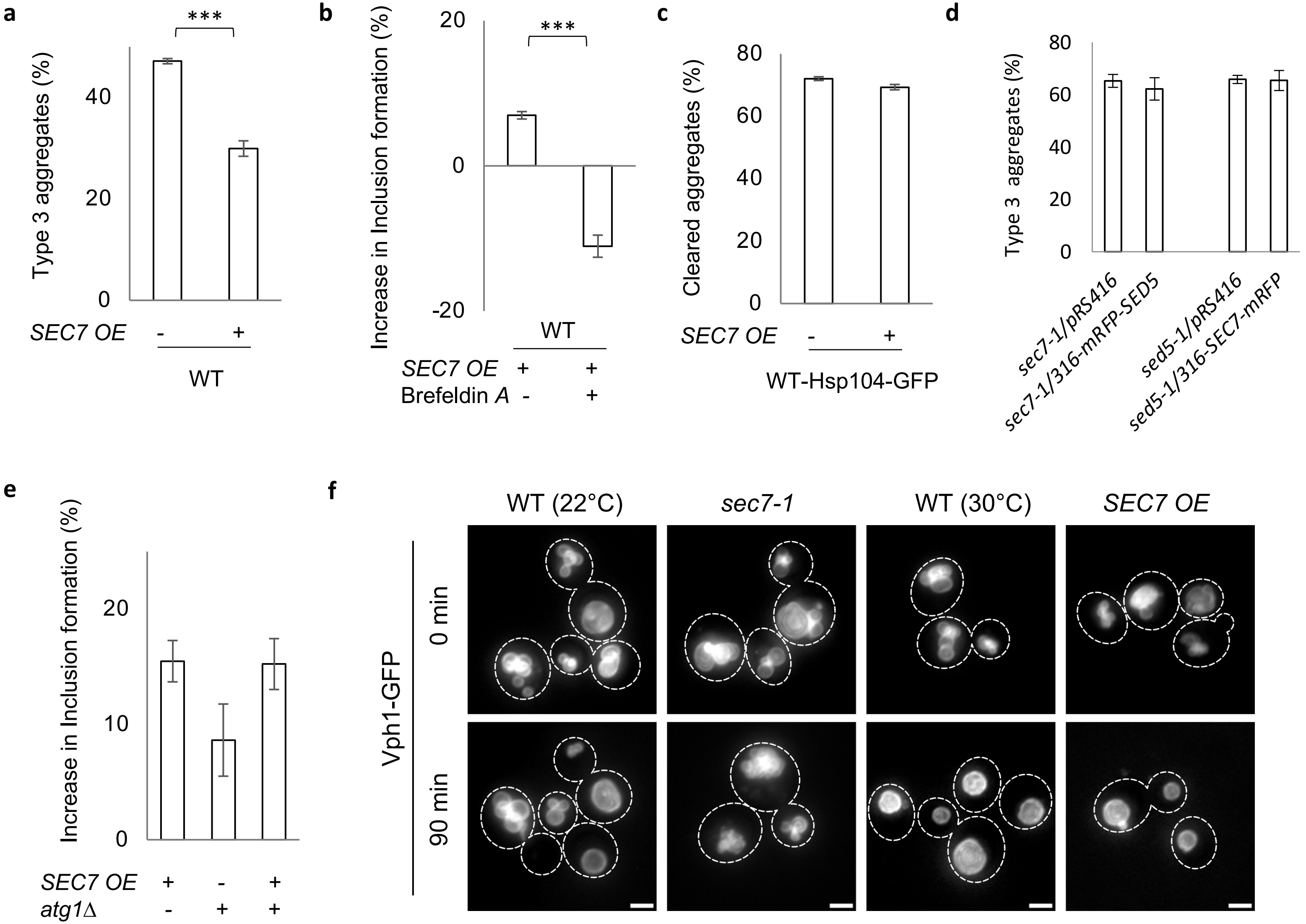

Supplementary figure 1: Sec7 overproduction boosts spatial PQC during heat shock.

A: Fraction of Hsp104-GFP type 3 upon continuous heat shock (38˚C, 90 min) in WT cells without and with overexpression of genomic *SEC7* via the *GPD* promoter compared to the WT.

B: Increase in inclusion formation of Hsp104-GFP aggregates after 90 min at 38° C of Sec7 overproducing cells (using 316-SEC7-mRFP plasmid (*ADH1* promoter)) with or without brefeldin A treatment.

C: Clearance of Hsp104-GFP foci in WT cells without and with overexpression of genomic *SEC7* via the *GPD* promoter after 1 hour heat shock at 38° C followed by recovery for 1 hour at 30° C.

D: Fraction of Hsp104-GFP type 3 upon continuous heat shock (38˚C, 90 min) in *sec7-1* or *sed5-1* cells expressing vector control (pRS416) or overexpressing *SED5* or *SEC7* via plasmids (*ADH1* promoter).

E: Increase in inclusion formation of Hsp104-GFP aggregates at 90 min at 38° C of WT cells with *SEC7* overexpression (*GPD* promoter) and without or with lack of *ATG1*.

F: Representative images of vacuoles (Vph1-GFP) in WT cells and *sec7-1* cells that were shifted from 22° C to 38° C for 90 min. Representative images of WT cells without and with overexpression of *SEC7* (*GPD* promoter) shifted from 30° C to 38° C for 90 min. Images are maximum Z-projections of relevant slices with brightness/contrast adjustment to visualize vacuoles. Scale bar 2μm.

Supplementary figure 2

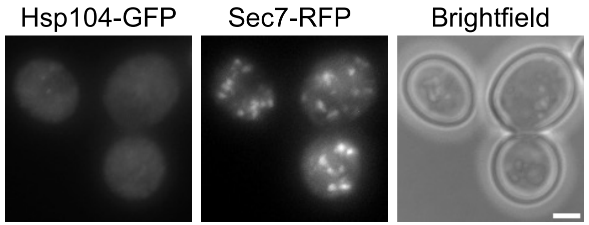

Supplementary figure 2: Hsp104-GFP and Sec7-RFP before heat shock in WT cells. Images are maximum Z-projections of relevant slices with brightness/contrast adjustment. Scale bar 2 μm.

Supplementary figure 3

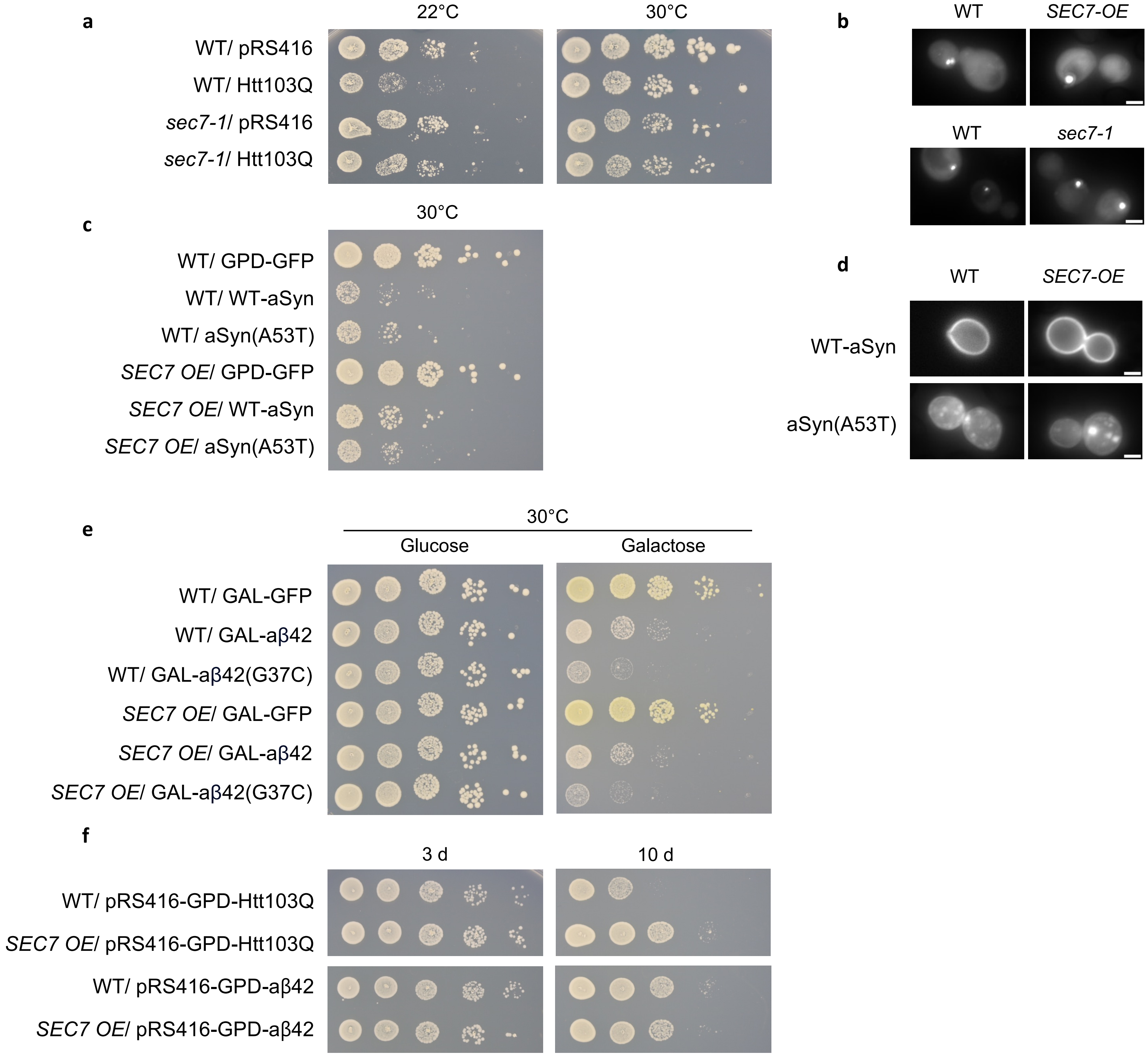

Supplementary figure 3: Overproduction of Htt103Q does not exacerbate fitness defects in *sec7-1* and cannot mitigate toxicity of toxic α-synuclein and amyloid-β constructs.

A: Fitness of WT and *sec7-1* cells overproducing Htt103Q via the *GPD* promoter (Htt103Q) compared to the vector control (pRS416) after 3 days of growth.

B: Htt103Q-GFP in WT, *SEC7 OE* and *sec7-1* cells at 30° C. Images are maximum Z-projections of relevant slices with brightness/contrast adjustments. Scale bar 2 µm.

C: Fitness of WT and *SEC7 OE* cells overproducing wild type and mutant α-synuclein via the *GPD* promoter compared to the vector control (GPD-GFP) after 3 days of growth.

D: Wild type and mutant constructs of α-synuclein-GFP in WT and *SEC7 OE* cells after galactose induction for 5 hours. Images of WT-aSyn are a single Z slice, images of aSyn(A53T) are maximum Z-projections of relevant slices. Images with brightness/contrast adjustments. Scale bar 2 µm.

E: Fitness of WT and *SEC7 OE* cells overproducing wild type and mutant aβ42 constructs via a galactose inducible plasmid compared to the vector control (GAL-GFP) after 3 days of growth on glucose and galactose.

F: Fitness of WT and *SEC7 OE* cells overproducing Htt103Q or aβ42 via the constitutive *GPD* promoter after growth in stationary phase at 30° C.- Cell suspensions were spotted after 3 days and 10 days and plates were kept at 30° C for 2 days.

Table S1: Hits found in the essential mutant yeast library by comparing mutants to control strains at 110 min heat shock.

Type 3 cells at 110 min, descending order of average number of aggregates per cell, cutoff=3.

ts allele	ORF
hsf1-848	YGL073W
fcp1-1	YMR277W
tfb1-1	YDR311W
cdc19-1	YAL038W
tfa2-45	YKR062W
spt5-194	YML010W
ceg1-ts	YGL130W
ccl1-ts4	YPR025C
ssl1-T242I	YLR005W
rna15-58	YGL044C
cdc25-1	YLR310C
sub2-1	YDL084W
ura6-6	YKL024C
rad3-ts14	YER171W
med6-ts	YHR058C
srb4-2	YER022W
pcf11-ts10	YDR228C
kar2-159	YJL034W
rgr1-100	YLR071C
tub4-Y445D	YLR212C
nut2-ts	YPR168W
med7-163	YOL135C
sec61-2	YLR378C
srm1-ts	YGL097W
rna1-S116F	YMR235C
mex67-ts5	YPL169C
bos1-1	YLR078C
med8-51	YBR193C
sec65-1	YML105C
yrb1-51	YDR002W
rpo21-1	YDL140C
hrp1-1	YOL123W
emg1-1	YLR186W
brl1-K405I	YHR036W
cdc31-2	YOR257W
stt4-4	YLR305C
glc7-12	YER133W
dbp5-2	YOR046C
abf1-103	YKL112W
srp102-510	YKL154W
prp19-1	YLL036C
cdc36-16	YDL165W
sec21-1	YNL287W
sec18-1	YBR080C
sly1-ts	YDR189W
sec9-4	YGR009C
rpt4-145	YOR259C
rpt6-20	YGL048C
sec16-2	YPL085W
ded1-F144C	YOR204W
gle1-4	YDL207W
sed5-1	YLR026C
cdc48-1	YDL126C
sec22-3	YLR268W
cog2-1	YGR120C
cog3-2	YER157W
sgt1-5	YOR057W
sec17-1	YBL050W
act1-108	YFL039C
sec23-1	YPR181C
mss4-102	YDR208W
sec31-1	YDL195W
ala1-1	YOR335C
rpn12-1	YFR052W
sec7-1	YDR170C
gdi1-1	YER136W
cmd1-8	YBR109C
yip1-4	YGR172C
rpt1-1	YKL145W
sup35-td	YDR172W
sec26-11D2(	YDR238C
crm1-1	YGR218W
dim1-2	YPL266W
sec2-41	YNL272C
tfb3-ts	YDR460W
cdc5-1	YMR001C

scc4-4	YER147C
gpi13-4	YLL031C
sec15-1	YGL233W
pik1-104	YNL267W
cdc39-1	YCR093W
cks1-35	YBR135W
rpn1-821	YHR027C
sec12-1	YNR026C
pfy1-13	YOR122C
pap1-1	YKR002W
yef3-F650S	YLR249W
pam18-1	YLR008C
rpn5-1	YDL147W
ypt1-3	YFL038C
sec62-ts	YPL094C
lst8-6	YNL006W
exo84-102	YBR102C
sec24-20	YIL109C
mcd4-174	YKL165C
cet1-15	YPL228W
med4-54	YOR174W
sec10-2	YLR166C
hyp2-1	YEL034W
dna2-2	YHR164C
vti1-2	YMR197C
uba1-1	YKL210W
sec5-24	YDR166C
mps3-7	YJL019W
nup145-R4	YGL092W
cdc37-ts	YDR168W
cop1-1	YDL145C
uso1-1	YDL058W
lcb2-1	YDR062W
cdc53-1	YDL132W
bet1-1	YIL004C
cof1-8	YLL050C

Table S2: Hits found in the essential mutant yeast library comparing 330 min to 110 min heat shock.

Percentage of Hsp104-GFP aggregates after continuous heat shock for 330 min compared to the initial time point, 110 min. Cutoff = 80%.

Ts allele	ORF	% Hsp104-GFP aggregates
spt14-1-10C	YPL175W	125.5
stt3-2	YGL022W	120.0
mps1-6	YDL028C	115.1
rgr1-100	YLR071C	103.4
srm1-ts	YGL097W	101.3
srp101-47	YDR292C	101.3
cks1-35	YBR135W	99.2
nup159-1	YIL115C	98.4
sec21-1	YNL287W	97.8
rpn6-1	YDL097C	96.2
rpt1-1	YKL145W	96.1
hsf1-848	YGL073W	96.0
sec65-1	YML105C	96.0
prp43-ts2	YGL120C	95.5
rna1-S116F	YMR235C	93.9
mss4-102	YDR208W	93.0
gpi19-2	YDR437W	92.8
mps3-7	YJL019W	92.7
rpt6-20	YGL048C	91.8
wbp1-1	YEL002C	91.4
arc35-6	YNR035C	91.4
sed5-1	YLR026C	90.7
cog3-2	YER157W	90.4
med8-51	YBR193C	90.3
rad3-ts14	YER171W	89.7
sec62-ts	YPL094C	89.2
gpi13-4	YLL031C	89.0
scd5-PP1D2	YOR329C	88.9
nse4-ts2	YDL105W	88.9
mex67-ts5	YPL169C	88.7
stt4-4	YLR305C	88.0
sec22-3	YLR268W	87.6
tfa2-45	YKR062W	87.4
apc11-22	YDL008W	87.1
scc4-4	YER147C	86.8
sec6-4	YIL068C	86.8
pik1-104	YNL267W	86.8
pan1-4	YIR006C	86.3
med11-ts	YMR112C	86.3
spt5-194	YML010W	86.2
hts1-1	YPR033C	86.2
gle1-4	YDL207W	85.9
sec23-1	YPR181C	85.8
kin28-ts	YDL108W	85.7
ura6-6	YKL024C	85.4
sgt1-5	YOR057W	85.4
sec63-1	YOR254C	85.3
sec16-2	YPL085W	85.3
cdc25-1	YLR310C	85.1
sly1-ts	YDR189W	84.7
rpn5-1	YDL147W	84.4
cdc8-2	YJR057W	84.2
tub4-Y445D	YLR212C	84.2
bet1-1	YIL004C	84.1
sec24-20	YIL109C	84.1
pam18-1	YLR008C	84.1
ypt1-3	YFL038C	84.1
sec5-24	YDR166C	84.0
ala1-1	YOR335C	83.9
exo84-102	YBR102C	83.6
spt6-14	YGR116W	83.0
sec7-1	YDR170C	82.9
sec26-11D2(	YDR238C	82.8
sec17-1	YBL050W	82.7
rpn11-14	YFR004W	82.7
sec31-1	YDL195W	82.7
ssl1-T242I	YLR005W	81.6
dpm1-6	YPR183W	81.1
cop1-1	YDL145C	80.9
sec9-4	YGR009C	80.8
arc15-10	YIL062C	80.6
uso1-1	YDL058W	80.4
rpt4-145	YOR259C	80.3
rpt3-1	YDR394W	80.2
pcf11-ts10	YDR228C	80.1
bos1-1	YLR078C	80.1

Table S3: Plasmids and strains with their genotype and origin used in this study.

Figure	Name	Genotype/Description	Origin
1B, C	WT (SGA)	*MATa his3Δ::kanMX4 can1Δ::STE2pr-Sp_his5 lyp1Δ ura3Δ0 met15Δ0 HSP104-GFP-LEU2*	This study
	sec7-1 (SGA)	*MATa his3Δ0 sec7-1::kanMX4 can1Δ::STE2pr-Sp_his5 lyp1Δ ura3Δ0 met15Δ0 HSP104-GFP-LEU2*	This study
1D, E	WT (SGA) with pRS416	*pCEN, URA3*	N/A
	WT (SGA) with 316-SEC7-mRFP	*pCEN, pRS316, SEC7-mRFP (ADH1 promoter), URA3 AmpR*	Kurokawa et al., 2014
2A	Same as 1D		
2B	ssa1D (SGA) with pRS416/316-SEC7-mRFP	*MATa his3Δ0 ssa1::kanMX4 can1Δ::STE2pr-Sp_his5 lyp1Δ ura3Δ0 met15Δ0 HSP104-GFP-LEU2*	This study
2C	ssa2D (SGA) with pRS416/316-SEC7-mRFP	*MATa his3Δ0 ssa2::kanMX4 can1Δ::STE2pr-Sp_his5 lyp1Δ ura3Δ0 met15Δ0 HSP104-GFP-LEU2*	This study
2D	Same as 2A, B, C		
2E	Same as 2A, B, C		
2F	Same as 2B		
2G	Same as 2C		
3A, B, C	BY4741 Hsp104-GFP-HIS3	*MATa his3Δ1 leu2Δ0 met15Δ0 ura3Δ0 HSP104-GFP-HIS3*	Huh et al., 2003
	BY4741 Hsp104-GFP-HIS3 NAT-pGPD-SEC7	*MATa his3Δ1 leu2Δ0 met15Δ0 ura3Δ0 HSP104-GFP-HIS3 pGPD-SEC7:NAT*	Per Widlund
3D	BY4741	*MATa his3Δ1 leu2Δ0 met15Δ0 ura3Δ0*	EUROSCARF
	BY4741 NAT-pGPD-SEC7	*MATa his3Δ1 leu2Δ0 met15Δ0 ura3Δ0 pGPD-SEC7:NAT*	Per Widlund
4A, B, S3F	BY4741 with pRS416	*pCEN, URA3*	This study
	BY4741 with pRS416-GPD-Htt103Q		Krobitsch et al., 2000
	BY4741 NAT-pGPD-SEC7 with pRS416	*pCEN, URA3*	This study
	BY4741 NAT-pGPD-SEC7 with pRS416-GPD-Htt103Q		This study, Krobitsch et al., 2000
4C	BY4741 with pRS416	*pCEN, URA3*	This study
	BY4741 with pRS416-GPD-aß42		Dina Petranovic
	sec7-1 with pRS416	*pCEN, URA3*	This study
	sec7-1 with pRS416-GPD-aß42		This study, Dina Petranovic
4D	BY4741 with pYX242		Babazadeh et al., 2019
	BY4741 with pYX242-WT-SYN		Babazadeh et al., 2019
	BY4741 with pYX242-SYN(A30P)		Babazadeh et al., 2019
	sec7-1 with pYX242		This study
	sec7-1 with pYX242-WT-SYN		This study
	sec7-1 with pYX242-SYN(A30P)		This study
S1A	BY4741 Hsp104-GFP-HIS3 NAT-pGPD-SEC7	*MATa his3Δ1 leu2Δ0 met15Δ0 ura3Δ0 HSP104-GFP-HIS3 pGPD-SEC7:NAT*	Per Widlund
S1B	Same as 1B		
S1C	Same as S1A		
S1D	sec7-1 (SGA) with pRS416 or pRS316-mRFP-SED5		This study, Babazadeh et al., 2019
	sed5-1 (SGA) with pRS416 or pRS316-mRFP-SEC7		This study, Babazadeh et al., 2019
S1E	BY4741 NAT-pGPD-SEC7	*MATa his3Δ1 leu2Δ0 met15Δ0 ura3Δ0 pGPD-SEC7:NAT*	Per Widlund
	atg1Δ	*MATa his3Δ1 leu2Δ0 met15Δ0 ura3Δ0 HSP104-HIS3 pGPD-SEC7:NAT atg1Δ::hphNT1*	This study
	SEC7 OE atg1Δ	*MATa his3Δ1 leu2Δ0 met15Δ0 ura3Δ0 HSP104-GFP-HIS3 atg1Δ::hphNT1*	This study
	Hsp104-GFP	*MATa his3Δ1 leu2Δ0 met15Δ0 ura3Δ0 HSP104-HIS3*	Huh et al., 2003
S1F	BY4741 Vph1-GFP-HIS3	*MATa his3Δ1 leu2Δ0 met15Δ0 ura3Δ0 VPH1-GFP-HIS3*	Huh et al., 2003
	SEC7 OE Vph1-GFP-HIS3	*MATa his3Δ1 leu2Δ0 met15Δ0 ura3Δ0 VPH1-GFP-HIS3 pGPD-SEC7:NAT*	This study
S2	Same as 2E		
S3A, B	BY4741 with pRS416	*pCEN, URA3*	This study
	BY4741 with pRS416-GPD-Htt103Q	*pCEN, URA3*	Krobitsch et al., 2000
	sec7-1 with pRS416	*pCEN, URA3*	This study
	sec7-1 with pRS416-GPD-Htt103Q	*pCEN, URA3*	This study, Krobitsch et al., 2000
S3C, D	BY4741 or with NAT-pGPD-SEC7 with pRS426-GPD-GFP	*2µ, URA3*	This study, Tiago Outeiro
	BY4741 or with NAT-pGPD-SEC7 with pRS426-GPD-GFP-aSyn	*2µ, URA3*	This study, Tiago Outeiro
	BY4741 or with NAT-pGPD-SEC7 with pRS426-GPD-GFP-aSyn(A53T)	*2µ, URA3*	This study, Tiago Outeiro
S3E	BY4741 or with NAT-pGPD-SEC7 with pRS426-GAL-GFP	*2µ, GAL inducible, URA3*	This study, Joris Winderickx
	BY4741 or with NAT-pGPD-SEC7 with pRS426-GAL-GFP-abeta42	*2µ, GAL inducible, URA3*	This study, Joris Winderickx
	BY4741 or with NAT-pGPD-SEC7 with pRS426-GAL-GFP-abeta42(G37	*2µ, GAL inducible, URA3*	This study, Joris Winderickx
S3F	BY4741 with pRS416-GPD-aß42		Dina Petranovic
	NAT-pGPD-SEC7 with pRS416-GPD-aß42		This study

References

Babazadeh R, Ahmadpour D, Jia S, Hao X, Widlund P, Schneider K, Eisele F, Edo LD, Smits GJ, Liu B, Nystrom T. Syntaxin 5 Is Required for the Formation and Clearance of Protein Inclusions during Proteostatic Stress. Cell Rep. 2019 Aug 20;28(8):2096-2110.e8. doi: 10.1016/j.celrep.2019.07.053. PMID: 31433985.
Huh WK, Falvo JV, Gerke LC, Carroll AS, Howson RW, Weissman JS, O'Shea EK. Global analysis of protein localization in budding yeast. Nature. 2003 Oct 16;425(6959):686-91. doi: 10.1038/nature02026. PMID: 14562095.
Kurokawa K, Okamoto M, Nakano A. Contact of cis-Golgi with ER exit sites executes cargo capture and delivery from the ER. Nat Commun. 2014 Apr 14;5:3653. doi: 10.1038/ncomms4653. PMID: 24728174; PMCID: PMC3996532.
Krobitsch S, Lindquist S. Aggregation of huntingtin in yeast varies with the length of the polyglutamine expansion and the expression of chaperone proteins. Proc Natl Acad Sci U S A. 2000 Feb 15;97(4):1589-94. doi: 10.1073/pnas.97.4.1589. PMID: 10677504; PMCID: PMC26479.
